# Learning from doing: the case for combining normalisation process theory and participatory learning and action research methodology for primary healthcare implementation research

**DOI:** 10.1186/s12913-016-1587-z

**Published:** 2016-08-03

**Authors:** Tomas de Brún, Mary O’Reilly-de Brún, Catherine A. O’Donnell, Anne MacFarlane

**Affiliations:** 1 Discipline of General Practice, School of Medicine, National University of Ireland, Galway, Ireland; 2 General Practice and Primary Care, Institute of Health and Wellbeing, University of Glasgow, Glasgow, UK; 3 Graduate Entry Medical School, University of Limerick, Limerick, Ireland

**Keywords:** Primary  healthcare, participatory learning & action research, Normalisation process theory, Implementation research, Theoretical frameworks

## Abstract

**Background:**

The implementation of research findings is not a straightforward matter. There are substantive and recognised gaps in the process of translating research findings into practice and policy. In order to overcome some of these translational difficulties, a number of strategies have been proposed for researchers. These include greater use of theoretical approaches in research focused on implementation, and use of a wider range of research methods appropriate to policy questions and the wider social context in which they are placed. However, questions remain about how to combine theory and method in implementation research. In this paper, we respond to these proposals.

**Discussion:**

Focussing on a contemporary social theory, Normalisation Process Theory, and a participatory research methodology, Participatory Learning and Action, we discuss the potential of their combined use for implementation research. We note ways in which Normalisation Process Theory and Participatory Learning and Action are congruent and may therefore be used as heuristic devices to explore, better understand and support implementation. We also provide examples of their use in our own research programme about community involvement in primary healthcare.

**Conclusions:**

Normalisation Process Theory alone has, to date, offered useful explanations for the success or otherwise of implementation projects *post-implementation*. We argue that Normalisation Process Theory can also be used to prospectively support implementation journeys. Furthermore, Normalisation Process Theory and Participatory Learning and Action can be used together so that interventions to support implementation work are devised and enacted with the expertise of key stakeholders. We propose that the specific combination of this theory and methodology possesses the potential, because of their combined heuristic force, to offer a more effective means of supporting implementation projects than either one might do on its own, and of providing deeper understandings of implementation contexts, rather than merely describing change.

**Electronic supplementary material:**

The online version of this article (doi:10.1186/s12913-016-1587-z) contains supplementary material, which is available to authorized users.

## Background

The development and implementation of research findings is not a straightforward matter. There are substantive and recognised gaps in the process of translating research findings into practice and policy [1, 2]. One well-recognised translational gap is between the development of new treatments and implementation of these in practice with the intended service users/patient or population groups, the so-called ‘know-do’ gap. Another relates to using the results of health services research to inform wider health-related policy and to influence research-policy-practice links [3, 4]. However, before such implementation reaches a stage of action, that is ‘doing’, the individuals and groups involved frequently have to go through a process of consideration about the nature and complexity of implementation work. It is important that individuals and groups engage in a comprehensive consideration of these issues so as to better inform their actions. However, often individuals and groups are not supported through this process. We refer to this as the ‘think-do’ gap in implementation projects.

In addressing these translational gaps, implementation researchers are encouraged to consider greater use of theoretical approaches [1, 5] and a more diverse and innovative range of methodological approaches from health services and community-based research, such as qualitative methods and participatory approaches [2–4] to improve the outcome of implementation projects. In addition, Davis et al’s systematic review of the use of theory in implementation research [6] suggests that researchers give careful consideration to their choice of theory and provide a rationale for that choice. Another key issue is to develop knowledge about how theories, generally designed as heuristic devices to stimulate thinking, can be *operationalised as action* in an implementation journey. For example, the success of guidelines is often limited when they are applied in primary care settings because of their disease orientation and lack of focus on patients’ and communities’ needs. Primary care could benefit from methods that involve people and ‘community centeredness’ in a systematic way, and this is where the combination of NPT and PLA might be of benefit [7]. This would build on the success observed in other fields when participatory methods and theoretical frameworks are brought together, for example in the implementation of e-health interventions [8, 9].

In this paper we respond to the above proposals. We consider, in brief, the current use of theory and diverse methods in the field of implementation science generally, before focussing on a combination of theory and method that we have experience of from our programme of research about community involvement in primary healthcare: Normalisation Process Theory (NPT) and Participatory Learning and Action (PLA). We will describe NPT and our rationale for its completed and planned use in our research. We will highlight the need to explore effective methodological partners, particularly for the prospective use of NPT and, from there, describe PLA, its relevance for implementation research and the apparent congruence between NPT and PLA for investigating and supporting the implementation of innovation in primary healthcare settings. We conclude that, taken together, they may offer a potent heuristic for prospective, implementation research, a heuristic with which to ‘think-do’ which may have greater potential to improve implementation processes than if they were to be used separately.

## Discussion

### The value of theory for implementation science

Eccles et al. [1] have argued that in order to overcome translational gaps in implementation research we need to see greater use of theoretical approaches, on the basis that this will offer (i) generalisable frameworks that can be applied across different settings and individuals, (ii) an opportunity for the incremental accumulation of knowledge, and (iii) an explicit framework for analysis.

Approaching this from our inter-disciplinary perspectives, we see theory as sets of assumptions or concepts, or a relatively abstract inquiry that is distinguished from empirical research or practical recommendations. At this ‘high’ level, theory is generally abstract and broadly applicable [10]. The term theory is, however, also used interchangeably with, and as an analogue for, model or framework [11, 12] particularly when it operates at the mid-level, namely where it is less abstract and addresses specific phenomena that can be translated into testable propositions [10]. In this situation, theory is understood as a heuristic device to ‘think through’ research questions, data acquisition and analysis. As with most applied empirically-based social science work, we believe that actual real life situations and contexts should inform theory making and theory use, so that theory may be enabled to speak effectively and usefully across a spread of different situations or clusters of settings that share sets of key characteristics [1]. We believe that if theory is developed, used and interrogated critically [13] so it doesn’t ‘flatten out’ the specificity and contextual factors encountered in different organisational settings, then it certainly has the scope to impact positively on implementation science [14].

There is growing interest in the use of cognitive, behavioural and organisational theories in implementation research [15]. There are a range of theories and conceptual frameworks in use that offer different foci for researchers, each offering a particular perspective, often influenced by the disciplinary background of the researchers who have developed and used it [16]. A recent review identified over 60 different theories or frameworks applicable to implementation research [12]. A more careful critique of these found that the focus varied from dissemination of research findings, through dissemination and supported implementation, to a principal focus on the conditions and actions required for implementation. Other approaches used in implementation research focus more on the organisational conditions than on the work required of individuals within the organisations. These include the *Diffusion of Innovations Theory* [17–19], *theories of collective and individual learning and expertise* [20–22], the *Theory of Reasoned Action* [23], and *Actor-Network Theory* [24, 25].

Each of these theoretical approaches has their own utility and value and it is not within the scope of this article to provide a comprehensive critique of each one. However, it is worth noting that the Theory of Reasoned Action lacks attention to social contexts and influences and, thus, has been criticised for being overly individualistic; Diffusions of Innovation Theory is excellent in understanding the introduction and spread of innovations but less helpful in terms of understanding the implementation work leading to routinisation in daily practice. Theories of collective and individual learning are overly focused on internalisation of innovations and Actor Network Theory is contentious because of its focus on inanimate objects and its lack of explanatory power [26]. In implementation research it is imperative that we understand how individual and collective action is required to implement new innovations and to understand more about how innovations become embedded in daily routine to such an extent that they become part of that daily routine – namely, how new ways of working become normalised. A recently developed contemporary social theory, Normalisation Process Theory, offers a comprehensive conceptual framework in this regard.

### Rationale for using normalisation process theory

NPT was developed as a response to multiple failures to implement innovations in complex healthcare contexts [15]. It is not a rigid model but is designed to emphasise the realities of implementation work in real time and space, and the inter-relationships between different kinds of implementation work. There are four components in NPT (Table 1): coherence (sense-making), cognitive participation (engagement), collective action (enactment) and reflexive monitoring (appraisal). Each of these has sub-components that can be used by researchers as sensitising concepts in implementation research [26–28]. A recent review [29] found that NPT has been used across a number of international settings and for analysis of implementation of innovations in primary and secondary healthcare settings. NPT is most frequently used retrospectively as an organizing framework for analyses and reporting findings. It has also been used to inform study/intervention design, to generate research questions for fieldwork and to create tools for investigating and supporting implementation [29]. The review concludes that NPT offers opportunities for incremental knowledge gain over time and an explicit framework for analysis, which can explain and potentially *shape* implementation processes. It was recommended that, in future NPT research, researchers should, where possible, involve multiple stakeholders including *service users* in order to enable analysis of implementation from a range of perspectives [29]. In terms of drawbacks or challenges associated with the application of NPT, the review notes that researchers involved in coding data under NPT constructs might have some difficulty with apparent overlaps between constructs. In an earlier paper [30], researchers noted some concern about correctly understanding the intended conceptual meaning of NPT constructs, as they wished to ensure that their analysis would be congruent with the theory. However, the benefits of using NPT clearly outweighed any such drawbacks.

**Table 1:** NPT constructs and sub-constructs

NPT Constructs			
Coherence	Cognitive Participation	Collective Action	Reflexive Monitoring
Can stakeholders make sense of the intervention?	Can stakeholders get others involved in implementing the intervention?	What needs to be done to make the intervention work in practice?	Can the intervention be monitored and evaluated?
Sub-constructs			
Differentiation	Enrolment	Interactional workability	Systematisation
Do stakeholders see this as a new way working?	Do the stakeholders believe they are the correct people to drive forward the implementation?	Does the intervention make it easier or harder to complete tasks?	Will stakeholders be able to judge the effectiveness of the intervention?
Individual specification	Initiation	Skill set workability	Individual appraisal
Do individuals understand what tasks the intervention requires of them?	Are they willing and able to engage others in the implementation?	Do those implementing the intervention have the correct skills and training for the job?	How will individuals judge the effectiveness of the intervention?
Communal specification	Activation	Relational integration	Communal appraisal
Do all those involved agree about the purpose of the intervention?	Can stakeholders identify what tasks and activities are required to sustain the intervention?	Do those involved in the implementation have confidence in the new way of working?	How will stakeholders collectively judge the effectiveness of the intervention?
Internalisation	Legitimation	Contextual integration	Reconfiguration
Do all the stakeholders grasp the potential benefits and value of the intervention?	Do they believe it is appropriate for them to be involved in the intervention?	Do local and national resources and policies support the implementation?	Will stakeholders be able to modify the intervention based on evaluation and experience?

We have chosen to use NPT in our research programme primarily for three reasons. First, because we are concerned with the *work* that individuals and groups have to do for a new technology or practice to become embedded and sustained in routine practice. Based on a social action approach to implementation, NPT has been developed through paying close attention to the concrete dynamics of actual implementation situations. From this, it has elucidated the individual and collective work involved in implementation processes. Second, it appeals to our sensibilities about theory because NPT is designed to be used as a heuristic device to critically interrogate data and we have used its precursor, the Normalisation Process Model, in this way retrospectively with good effect in prior research [31, 32]. Finally, we are particularly interested in the idea of using NPT prospectively to shape implementation processes and involve service users in the analysis of implementation processes [29]. The application of NPT to the prospective collection of data raises the opportunity to critically interrogate the usefulness of NPT and to explore what unique perspectives service users may have for analysing implementation processes.

We are therefore interested in advancing knowledge and understanding about the ways NPT might be applied or ‘operationalised’ in practice to support implementation work, particularly when its components refer to dynamic, complex and inter-related actions and interactions between individuals and organisational structures that are not open to manipulation. This specific challenge for NPT relates to the more general literature about the need for effective methodology to explore and support implementation research.

### Operationalising NPT in implementation research: the need for an effective methodology to explore and support implementation science

Randomised controlled trials (RCTs) are regarded as a gold standard in the domain of health services research focussed, as they are, on the question of *“does this intervention work”*? While explanatory trials measure efficacy, i.e. the benefit a new treatment or intervention can produce under ideal conditions, pragmatic trials answer questions of effectiveness, namely the benefit of a new treatment or intervention in the ‘real’ world [33]. Even then, pragmatic trials are often less sensitive to the dynamics of healthcare settings as complex systems that are characterised by non-linear interactions [34, 35] shaped by organisational culture.

Social and cultural anthropologists have researched societies, groups and organisations for over 100 years in an effort to better understand ‘culture’. One of the insights gained from that research is that much of what we call culture is *non-linear*, *shadowed*, and deeply embedded in the realm of the *non-rational;* expressive of *differing notions of what constitutes rational thinking and behaving, including differing rationalities* [36–38]. The issue of culture is germane here in that a number of authors within the implementation science domain have alluded to the fact that implementation settings are complex adaptive systems that have the capacity to self-organise, and that they have shadow systems within them that inhibit implementation [39–41]. These shadow systems are often poorly understood. We acknowledge that in implementation contexts we frequently have different cultural influences and rationalities layered onto and embedded within organisational cultures and rationalities. For example, primary care research relies on professions with diverse cultural norms, expectations and values, where there may or may not be the experience or structures to support professionals in their work together as inter-disciplinary teams. This is further complicated in that primary care professionals need to respond to culturally diverse communities, aiming to include all populations in their research, a core value of primary healthcare research. Implementers often fail to recognise and acknowledge these inter-related realities, leading to implementation failure. NPT draws attention to both individual and collective views of implementation, but is not itself a methodology. It therefore requires to be operationalised through appropriate methodological approaches.

Researchers need to develop methods and approaches that allow them to explore organisational cultures and settings as adaptive systems where the *non-rational* or *different rationalities* play a significant part in implementation in healthcare settings. The authors are fully aware that the concept of ‘culture’ is a highly contested one and a difficult and complex area to approach for the non-specialist. Researching culture, therefore, ideally requires the specialist skills and background of social scientists as part of multi-disciplinary research teams in implementation research. This is particularly important in primary healthcare which operates in communities where cultural diversity is an important and dynamic characteristic.

To meet this aim of description, exploration and analysis of culture in implementation research, there is, therefore, an obvious need to utilise qualitative research methods, and in particular ethnographic-style research. These are appropriate tools for uncovering some of the non-rational/differently rational and culture-related dimensions inherent in implementation situations and contexts. Brown and McCormack highlight that, in a previous ethnographic study they conducted [42] the use of ethnography was successful in identifying contextual issues that needed to be addressed or changed but provided *no opportunity to enact change* [40]. This means that, while deploying research methodologies that help us understand the shadow side and complexities of organisations is important and necessary, for implementation to actually work such ‘understanding’ only takes us so far. We need to consider methodologies that offer not only the possibility of understanding and deepening our knowledge, but also provide a mechanism for exploring and facilitating the enactment of change [43] – the *doing* side of the ‘know–do’ gap.

Therefore we propose that, in conjunction with other qualitative research methodologies including ethnographic research, implementation research might profit from enlisting the assistance of participatory and action research methodologies as a means of more effectively understanding and supporting the ‘know–do’ gap and implementation work. This view is supported by Greenhalgh et al.’s important review of the diffusion of innovations in service organisations [15] where one of the recommendations for future research was that implementation research should be participatory, engaging ‘on-the-ground’ practitioners as partners in the research process:

*‘Because of the reciprocal interactions between context and program success, researchers should engage ‘on-the-ground’ service practitioners as partners in the research process. Locally owned and driven programs produce more useful research questions and data that are more valid for practitioners and policymakers’.* ([15], pp 581-562)

Below we focus on one such methodology – Participatory Learning and Action (PLA) research.

### Participatory Learning and Action (PLA) research

PLA is a form of action research. It is a practical, adaptive research strategy that enables diverse groups and individuals to learn, work and act together in a co-operative manner, to focus on issues of joint concern, identify challenges, and generate positive responses in a collaborative and democratic manner [44, 45]. Its roots are in the global south where it was explicitly designed to address the introduction of planned change in international development contexts. Robert Chambers identified a serious ‘gap’ in development research where local people were usually missing from the stakeholder table and many internationally funded projects were failing to achieve their intended aims [46]. Chambers worked with communities to develop Participatory Rural Appraisal (PRA), a research approach that included and privileged ‘local experts’ (e.g. community representatives/service users/patients) as key stakeholders in research. Much of Chambers’ work was applied to the field of rural development. Under modernisation theory, which was the dominant international development theory at the time, local people (the so-called beneficiaries of change) were considered to be *the* factor in change situations that constituted a barrier to introducing an innovation. They were considered to be backward, largely uneducated and overly traditional in their thinking; all features that putatively impeded the implementation of change and innovation. PRA was successful in helping to deconstruct the conceit within modernisation theory that local populations are the problem, and refigured them as a resource for research and development projects – experts in their own right, along with those others more commonly identified as experts (for example, development professionals, service providers and planners). Influenced by Chambers’ work, two of the authors of this paper (de Brún and O’Reilly-deBrún) pioneered, over the past 25 years, the adaptation and application of participatory approaches and techniques to other fields of research in international development throughout sub-Saharan Africa (gender, development education, female education) and in Europe, to intercultural and migrant health. We use the term Participatory Learning and Action (PLA) to describe our work [47].

The healthcare literature contains many examples of a broad, growing family of ‘bottom-up’ participatory research approaches. This growing family is not a monolithic body of ideas and methods but a pluralistic orientation to knowledge-making and social change, and this is their overarching connection. A core shared principle is the inclusion of ‘local experts’ as active participants who provide their unique insights to the research endeavour. These approaches include, among others, Participatory Research (PR) [48–50] Participatory Action Research (PAR) [51–53] Emancipatory Action Research (EAR) [54, 55] Community Based Participatory Research (CBPR) [56–59] and Participatory Learning & Action (PLA) [47, 60]. Major reviews and comparisons of a wide range of participatory approaches and methods are also available in the literature [54, 61, 62]. Critiques of participatory methodologies alert us to important challenges, for example, the need to see community participation as a long-term process of implementation and support for improved health outcomes [50]; the need to develop funding streams for participatory research to ensure that local health priorities influence the research agenda [52] and the fact that many professional health researchers may be unprepared for the reversals of power and hierarchical relationships a participatory approach may require [49]. Notwithstanding these challenges, the great strength of participatory approaches lies in the democratic inclusion of locals as ‘experts in their own right’ – the reconfiguring of locals as stakeholders capable of providing unique insights to healthcare research. PLA is noteworthy in that it employs a range of highly-visual research techniques which are readily accessible to diverse stakeholder groups where asymmetries of power may exist [60]. PLA, when well-facilitated, ‘levels the playing field’ and the transparently democratic processes and techniques help to balance power differentials (e.g., within multi-disciplinary teams in primary care settings). To understand the potential of PLA to balance asymmetries of power, it is important to understand a series of key reversals [45, 47, 63–65] of attitude and practice that underpin its use in research and development projects (Table 2). PLA also highlights the ways that *learnings* are achieved across such diverse groups, and where and how such learnings are oriented towards co-designed *action planning* and implementation. For example, in previous work, we successfully used PLA to develop a multi-perspectival guideline to support cross-cultural communication in primary healthcare consultations. We formed a combined community-university research team, including seven established migrants whom we trained as peer PLA researchers. Their diverse linguistic abilities, cultural backgrounds and PLA training enabled them to safely access and meaningfully engage with hard-to-reach migrant service-users from six different language groups, representing diverse cultural and ethnic backgrounds. A significant proportion of the migrant service-users had low literacy, and our use of a wide range of PLA techniques enabled them to participate effectively in the research process, allowing their perspectives *and* those of health service-providers to come together in a PLA dialogue which resulted in a democratically generated and agreed guideline for cross-cultural communication. PLA was instrumental in generating safe spaces and sustained engagement of stakeholders over a two-year period and linked a hard-to-reach population with the academy and service-providers in a positive, productive community-university partnership for primary healthcare research [60, 66].

**Table 2:** Key reversals in participatory learning & action research methodology

Reversal from…	to
Assuming knowledge…	exploring and exchanging complex ‘knowledges’
Hierarchical relationships among stakeholders…	reciprocal and mutually empowering relationships
Viewing stakeholders as passive beneficiaries…	viewing stakeholders as active partners and collaborators who benefit differentially from research outcomes
Viewing stakeholders as problem makers…	to engaging with them as problem-solvers

PLA is highly relevant for the field of implementation research because it employs a pragmatic, multi-perspectival research methodology. We would argue that PLA is relevant to the field of implementation research because of its iterative and organic nature, and the ways it encourages diverse stakeholders to engage in cycles of research, co-analysis, reflection and evaluation over time. This highly reflexive process enables stakeholders to address issues of joint concern creatively in order to arrive at positive strategies to achieve goals, implement agreed actions and influence national and/or local policy [67]. Importantly, possible solutions to problems can be considered heuristically – ‘tried out’ and ‘fine-tuned’ through various iterations towards workable and sustainable outcomes for all stakeholders.

### NPT and PLA: working together to incrementally increase knowledge in implementation contexts

NPT and PLA both have relevance, as theory and method respectively, for the field of implementation research [68]. Through NPT’s framework, the various layers of activity and work inherent in innovation and implementation can be made explicit and available for investigation. NPT can enhance researchers’ knowledge of the implementation process, shaping data generation and analysis, and providing conceptual density [39]. However, as mentioned earlier, that alone may not be sufficient to support the *action* required to achieve implementation, i.e. to make it happen. It is interesting to think about closing the ‘know-do’ gap between knowing about implementation processes/problems and doing something about them. Through PLA’s specific problem-solving orientation, attention is paid to generating knowledge and understanding of implementation processes as well as the actions required for implementation. PLA is interested in engaging social actors to identify and report problems, and to work individually and collectively to identify potential solutions to those problems. In this way, PLA offers a significant added dimension in supporting implementation processes and exploring the enactment of implementation through PLA dialogue.

As well as their scope to impact on the field separately, we are interested in the ways in which there is congruence between NPT and PLA. In terms of their origins, PLA and NPT are congruent in that they have both been developed in response to multiple failures to implement innovations (albeit in quite distinct settings and contexts). Moreover, as we highlight here, NPT and PLA are epistemologically compatible because both are located within the broad social science constructivist paradigm, which acknowledges that reality is defined, and meaning is conveyed for members of groups and organisations, through a range of socio-cultural means. Such socio-cultural factors can be both explicit (mission statements, sets of formal rules, sets of guidelines) and implicit (‘non-rational’ and non-linear influences) [35, 39–41]. Given this congruence, we are interested in the ways a complementary partnership between NPT and PLA may offer exciting possibilities to better understand and support, theoretically and practically, the dynamics and pragmatics of implementation processes.

When we consider areas where NPT and PLA may lack congruence, we note that NPT has focused in the main on the perspectives of ‘professionals’ (service-providers, planners, policy-makers). This meant that service-users’ unique perspectives were missing from analysis of implementation processes [29]. Also, NPT does not explicitly consider issues of inclusivity and power. This potential drawback may, however, be balanced by PLA’s emphasis on inclusion of the least powerful at the stakeholder table. Conversely, a potential drawback of PLA may be that being rooted so strongly in the experiential world of stakeholders, a PLA research process might ‘miss’ important implementation questions or topics that NPT alerts us to. Taken together, then, NPT and PLA may enhance each other, offering a potentially stronger heuristic than either on their own might otherwise provide. Such a heuristic can help researchers to explore more adequately ‘think-do’ gaps in the emerging field of implementation science, and also have a positive impact on the ‘know-do’ gaps that are currently evident in practice and policy.

Below, we provide a concrete example (Table 3) of the use of NPT and PLA in the development of a framework for implementation of community participation in primary healthcare [68]. NPT constructs and sub-constructs (Table 1) alert researchers to a range of important factors that promote or inhibit implementation. For the Framework, NPT was used to develop a comprehensive set of research questions designed to enhance understanding (retrospectively) about what affected implementation processes in a variety of community participation projects in primary healthcare across rural and urban locations in Ireland [68]. For example, the NPT construct ‘collective action’ which relates to ‘enactment’ or ‘getting the work done’ generated research questions about stakeholder involvement: was it tokenistic or meaningful? Were adequate resources and skills available to stakeholders to enable them to do the implementation work meaningfully? Using a PLA approach and techniques, researchers explored these and other NPT-derived questions during data generation and empirical fieldwork with diverse stakeholders. This combined use of NPT and PLA ensured that all NPT constructs were covered and that a range of stakeholders’ perspectives on these key implementation issues were elicited and represented. Key benefits of this application of NPT and PLA included (a) a more developed understanding of the complex interplay that existed between individual, organisational and social factors, (b) clarification of the ideal conditions for implementing community participation in primary healthcare in the Irish primary care context and (c) a set of recommendations which, given the use of NPT as a theoretical framework, may have transferability and relevance across a variety of contexts for projects that have, as their core objective, meaningful community involvement. The Framework is an example of the use of NPT as a heuristic device to stimulate thinking and of PLA as the methodological process through which ‘NPT thinking’ was operationalised as ‘action’ with stakeholders: stakeholders identified levers and barriers to implementation and participated in the development of recommendations for future action to ensure meaningful community participation. It is this interplay between NPT and PLA which allows their combined heuristic potential to unfold. Essentially, NPT is a theory capable of supporting the individual or group to think through issues involved in implementation, thereby addressing the ‘think-do’ gap. PLA offers a methodology capable of translating thinking into concrete action – operationalising ‘thinking’ into ‘doing’/action.

**Table 3:** Community participation in primary healthcare: identifying actions to improve implementation using normalisation process theory and participatory learning and action research

NPT Construct	NPT informed questions re community participation in primary healthcare explored in PLA fieldwork	Problems in the practice of community participation in primary healthcare identified with stakeholders during PLA fieldwork that impact on implementation	Recommended actions
Coherence	How is service user involvement defined?	Multiple terms are in use. People use the same terms to mean different things. There is lack of shared understanding about the work involved across stakeholders.	All stakeholders clarify their own understanding of community participation in primary healthcare and, through dialogue with each other, arrive at a shared understanding of community participation in primary healthcare with other stakeholders at the start of a community participation project.
Cognitive Participation	Why do stakeholders get involved?	There is a lack of clarity about why different stakeholders get involved. People get involved for different reasons and there is a lack of understanding about the roles that people play.	Stakeholders work together to clarify who needs to be involved and agree to work together to drive the implementation of a community participation in primary care project forward.
Collective Action	What methods are used?	Involvement can be tokenistic. There is often a lack of adequate resources and skills to do the work meaningfully. Stakeholders are not clear about their individual roles.	All stakeholders should have appropriate organisational support, skills and training, trust in the work and the ability to perform all tasks involved in order to make an activity or process take place.
Reflexive Monitoring	What are the outcomes?	It is difficult to evaluate the impact of community participation in primary healthcare. Evaluation is often ad hoc and/or anecdotal.	Stakeholders will appraise their work, using formal and informal systems, so that they can learn about what is working well and can modify work practices to maximize community participation in primary healthcare.

Adapted from: MacFarlane, A., Tierney, E. and McEvoy, R. (2014) *A Framework for Implementation of Community Participation in Primary Care*; A University of Limerick and Health Service Executive Collaboration. 2014

### Engaging social actors in complex adaptive systems

In its commitment to investigating organisations as whole systems, or complex adaptive systems, NPT is rooted in the need to attend to the role of social actors, in terms of individual and collective action, in any organisation where change or innovation is to be introduced. As mentioned above, working with the four NPT constructs encourages researchers to pay attention to the full range of actors involved in implementation work and the nature of their reciprocal, interdisciplinary and inter-professional relationships [26, 27, 31]. Similarly PLA, influenced as it has been by social and cultural anthropology [44], understands the methodological need to approach organisations holistically and/or dialectically, paying due attention to the full range of social actors (participants/stakeholders) within organisations charged with implementing innovations, and to exploring complex social and cultural processes. Through a PLA dialogue, key stakeholder groups are encouraged to listen to, and learn from, each other’s knowledge and perspectives. Trust, rapport and mutual respect builds up in the early stages of engagement and this supports the ongoing cycles of work (i.e. research, co-analysis, reflection and evaluation) [60].

PLA also encourages stakeholders to be questioning of, and exercise control within their context as far as is practically possible – to think and reflect about the ways PLA processes and other stakeholders’ perspectives and knowledge can be used to challenge the seemingly ‘fixed’ elements of the ‘real world’ of their particular organisational setting. For example, one way of facilitating this might be to invite stakeholders to imagine ideal solutions and scenarios that could challenge or change their organisational context, if this is deemed desirable by them in terms of implementing an innovation.

PLA thus promotes the active engagement of all stakeholders in such processes and can support actors to make even small ‘transformative leaps of generosity’ towards each other and away from familiar and ‘precious’ territorialities that can inhibit implementation [47, 69]. Building trust is a necessary prerequisite in creating the conditions for such leaps of generosity to occur. Brown and McCormack [40] allude to the importance of creating psychologically safe spaces through the use of an action research methodology in the effective management of pain among older people. Given that PLA is broadly a form of emancipatory research [70, 71] it explicitly seeks to articulate the voice of marginalised or underserved groups, communities and populations.

However, we should not forget that PLA also has the power to engage in an emancipatory way with other more professionally-orientated stakeholder groups in implementation settings, in terms of creating ‘conditions of safety’ within which to explore issues of professional territoriality, and other areas of tacit knowledge and practice that can inhibit implementation of innovation. This is illustrated in research carried out with homeless men in Dublin’s inner city in 2008 [69]. In addition to generating culturally appropriate knowledge about the experience of social exclusion among homeless men, and surfacing culturally appropriate solutions to that experience, the PLA process had the added and unintended outcome of galvanising the men as a group to begin the development of a new form of service provision offered by themselves to other homeless men in Dublin. The group is known as MAIN (Men Alone In No-man’s land) and the PLA process was the catalyst for these men to rethink their self-perceptions as relatively powerless individuals towards becoming a resource for other homeless men in inner-city Dublin. Through the experience of the PLA research, the men of MAIN began a process of recasting and rethinking their ascribed role as service users towards the more innovative and powerful one of service provider. To enable them to achieve this transformation they sought and received the support of statutory and non-statutory agencies to begin an outreach programme among their peers.

We propose that this heuristic possibility of PLA is not merely confined to the service user group as has so far been discussed in this illustration, but also to other key stakeholder groups. The key challenge for the commissioning agency in the example above was to allow its own practice and thinking to be touched by this heuristic possibility. As a statutory service provider organisation it was more used to receiving sets of recommendations from researchers that would clearly indicate what the provider organisation needed to do in order to answer the needs of the men of MAIN. What it found new and somewhat disconcerting, at least initially, was the shift in its organisational self-perception that was called for by the men in the PLA research process. A brokered conversation took place, through the researchers, between the men of MAIN and the service provider organisation. This eventually led to the organisation re-thinking at least some aspects of how it normally considered and practiced the delivery of services to homeless men in Dublin’s inner city. This balance of attention to all social actors and stakeholders as well as organisational cultures is crucial, because for change to occur in culturally distinct organisations the creation of safety for all is a precondition that allows for the possibility of a transformational moment occurring.

### Exploring implementation as a reflexive engagement

In keeping with their underlying epistemological stance, both NPT and PLA emphasise the importance of co-assessments and reflexive processes for all stakeholders engaged in implementation work. The NPT construct ‘Reflexive Monitoring’, is about reflecting on the implementation work itself, monitoring how the work is assessed by different stakeholders, tracking progress, and monitoring the effect of the implementation. PLA is essentially a sustained reflexive process that is rooted in the experience of all stakeholders in terms of monitoring and evaluating the detail and overall effects of any innovation. PLA dialogues can be used to promote and foster self-reflection and self-evaluation as well as bringing differing knowledges and perspectives into conversation with each other. Related to this, PLA is always inherently about the generation of emic perspectives and approaches where insider knowledge is valued and surfaced [72, 73].

PLA researchers/practitioners can engage with key stakeholders to enable what is often expert but implicit knowledge to become explicit and therefore available for exploration and analysis in an ongoing and respectful dialogue. Emic perspectives were developed by cognitive anthropologists as a way of accessing deep cultural knowledge through linguistic analysis [72], and therefore lend themselves to helping surface some of the already mentioned seemingly non-rational or shadow systems within individual organisational cultures. However, precisely because of PLA’s interest in bringing different forms of knowledge into articulation with each other across stakeholder groups, it can also acknowledge the role and importance of knowledge generated in an etic manner that either informs, or emerges during PLA dialogues. Examples of ‘etic knowledges’ will include those of different professions who have a stake in implementation, including researcher knowledge and community-based forms of knowledge; though we point out that professionals and researchers also possess emic forms of knowledge. Paying attention to both emic and etic perspectives may be particularly useful in fostering conversations in implementation settings between diverse stakeholders whose differing knowledges and perspectives are brought to the fore by the use of the theoretical framework. PLA approaches can help interrogate the theory, to identify which elements of the theory are relevant to a specific context or setting, and which elements of the implementation work in that specific context or setting may not be addressed or covered by the theory.

This attention to, and management of, etic and emic perspectives requires a specific attitudinal disposition on the part of the researcher/participatory research practitioner towards stakeholders, which is based on the key reversals involved in PLA and underpins a PLA mode of engagement. In the context of bringing together NPT and PLA so as to better understand implementation in diverse contexts we emphasise the crucial importance of providing high quality training for researchers in PLA research methodology as well as in NPT.

Importantly, the use of PLA approaches has the potential to ensure that emic perspectives are not ‘lost’ through the use of NPT as an etic theoretical framework. NPT and PLA used together could help ‘keep in touch’ with the changing nature and dynamics of implementation work as defined and shaped by those involved in the work, using theory to explain, understand and support that work.

This approach is underpinning new research into the implementation of guidelines and training initiatives designed to support cross-cultural communication in primary care. Additional file 1: Figure S1 highlights an example of our current research, the RESTORE project (**RE**search into implementation **ST**rategies to support patients of different **OR**igins and language background in a variety of **E**uropean primary care settings) [74] which was conducted across five European primary care settings. The involvement of migrant service-users, along with other key stakeholders (general practice staff, primary care nurses, community interpreters, healthcare service planners) was core to the participatory design of RESTORE and a key component of the rationale for combining NPT and PLA was that both are inherently adaptive and responsive to diverse primary healthcare contexts. A PLA dialogue was developed around NPT constructs to prospectively inform the implementation of guidelines and/or training initiatives to support communication in cross-cultural general practice consultations. Essentially, RESTORE is the empirical ‘testing-ground’ for the proposals in this paper about the potential combined heuristic force of NPT and PLA to offer a more effective means of supporting implementation projects than either one might do on its own. RESTORE presents the opportunity to explore how to operationalise NPT and PLA prospectively and to ask in what ways NPT and PLA proved to be congruent or lacking in congruence. RESTORE may also provide insights about what it may mean, practically and methodologically, to use both NPT and PLA at the same time. How does it change data collection and analysis? This analysis is underway and results will be reported in separate papers. A detailed description of the study protocol has been published and is available [75].

## Conclusions

Implementation researchers are interested in bridging the ‘know-do’ gap. For this, we need to develop implementation strategies that are up to the task of enhancing knowledge and enacting change. Building on calls for the use of theory and action-oriented methodologies, this paper advances knowledge in the field of implementation research by exploring the combination of theory and method. We argue that NPT is a unique and sophisticated heuristic device for understanding and supporting implementation work. PLA is an action-oriented research methodology that also possesses heuristic power. Taken together, they potentially present an effective and potent heuristic to ‘think-do’ with. We shall monitor carefully the impacts and implications of testing this combination in our ongoing empirical work in the RESTORE project. We note that NPT on its own has, to date, been successful in offering explanations for why implementation projects have or have not worked *post*-implementation. Combining NPT and PLA also offers the possibility of prospectively demonstrating the effectiveness of this combination to actively foster change in implementation settings, rather than merely describing or commenting on change. This combination of theory and method also places us in a better position to continually refine and develop the theory.

Finally, we note that there may be potential for a broader view and practice of collaborative learning communities or networks that go beyond single implementation studies. For daily practice, NPT and PLA could serve as a method for primary health care to plan and review its responsiveness to community priorities. Its application in this context might benefit from an adaptation of the methodology to the time and staffing constraints of the health system. In addition, while potential challenges might emerge regarding different foci and priorities of research and health service and systems, we might also look toward establishing, over time, implementation science organizational research cultures. Here, researchers and researched could occupy a shared platform, with equal status and power to participate in the full continuum of research and research utilization.

## Abbreviations

FUSION, Framework for implementation of USer InvOlvemeNt in Primary Care; MAIN, Men Alone In No-man’s land; NPT, Normalization Process Theory; PLA, Participatory Learning and Action; PRA, Participatory Rural Appraisal; RCT, Randomized Control Trial; RESTORE, REsearch into implementation STrategies to support patients of different ORigins and language background in a variety of European primary care settings

## Additional file

Additional file 1: Figure S1. Combining NPT and PLA to involve migrant service users and other stakeholders in the implementation of guidelines and/or training initiatives to support communication in cross-cultural general practice consultations in five EU countries: The RESTORE project. (DOCX 13 kb)
