# Supplementary material for: Learning from doing: the case for combining normalisation process theory and participatory learning and action research methodology for primary healthcare implementation research
Source: BMC Health Serv Res. 2016 Aug 3;16:346. doi: 10.1186/s12913-016-1587-z (PMC4972957; doi:10.1186/s12913-016-1587-z)
Supplement: Additional file 1: Figure S1. — Combining NPT and PLA to involve migrant service users and other stakeholders in the implementation of guidelines and/or training initiatives to support communication in cross-cultural general practice consultations in five EU countries: The RESTORE project. (DOCX 13 kb) [file 12913_2016_1587_MOESM1_ESM.docx]

**Supplementary file**

**Figure 1: Combining NPT and PLA to involve migrant service users and other stakeholders in the implementation of guidelines and/or training initiatives to support communication in cross-cultural general practice consultations in five EU countries: The RESTORE project**

- RESTORE is concerned with optimising the delivery of primary health care to European citizens who are migrants who experience language and cultural barriers in host countries. We focus on the implementation of evidence-based health information (e.g. guidelines to enhance communication in cross-cultural consultations) and interventions (e.g. training initiatives on interculturalism and the use of paid interpreters) designed to address language and cultural barriers in primary care settings.
- In RESTORE, NPT and PLA are being used in combination with each other as a ‘know-do’ tool to investigate and support this implementation work. We will facilitate a PLA-brokered dialogue with key stakeholders – NGOs and service users from migrant communities, general practice staff, interpreting and/or cultural mediator staff, service planners and policy makers in five European countries: Austria, England, Greece, Ireland, and The Netherlands.
- The PLA-brokered dialogue is being developed as a mode of engagement whereby relationships of trust, mutual respect and rapport are fostered and encouraged within and across stakeholder groups so that data can be generated to elicit stakeholder explicit and hidden ‘knowledges’ for discussion and co-analysis.

We will facilitate a PLA-brokered dialogue to operationalise the NPT constructs described below:

1. **Coherence** – whether evidence-based health information and/or interventions designed to address communication in cross-cultural general practice consultations ‘make sense’ (or not) to stakeholders and whether they see the point of them (or not) for their work.
2. **Cognitive Participation** – whether there is engagement from stakeholders with some/one of the guidelines and/or training initiatives and whether there is ‘buy-in’ to actually engage in an implementation project.
3. **Collective Action** – participants’ experience of implementing their chosen guidelines and/or training initiative in their local setting with a focus on co-designing potential solutions to arising problems that can be tested by participants. Taken together, these two tasks create an iterative loop between analysis of initial experiences with the intervention and the work related to its implementation, exploration of potential solutions to any experienced problems, and testing of identified potential solutions by participants.
4. **Reflexive Monitoring** – mapping out how participants themselves appraise the implementation they have engaged with in their local settings and exploring the ways in which experiences of implementation work may shape and re-shape ‘coherence’, ‘cognitive participation’ and ‘collective action’.
